# Differences in cancer incidence and pattern between urban and rural Nepal: one-year experience from two population-based cancer registries

**DOI:** 10.3332/ecancer.2021.1229

**Published:** 2021-05-11

**Authors:** Ranjeeta Subedi, Atul Budukh, Sandhya Chapagain, Pradip Gyanwali, Bishal Gyawali, Kopila Khadka, Chanda Thakur, Uma Dahal, Rajesh Dikshit, Anjani Kumar Jha, Meghnath Dhimal

**Affiliations:** 1 Nepal Health Research Council, RamshahPath, Kathmandu 44600, Nepal; 2 Homi Bhabha National Institute, Tata Memorial Centre, Mumbai 400012, India; 3 National Academy of Medical Sciences, Bir Hospital, Kathmandu 44600, Nepal; 4 Departments of Oncology and Public Health Sciences, Division of Cancer Care and Epidemiology, Queen’s University, Kingston K7L 3N6, Canada

**Keywords:** cancer, incidence, registry, Nepal, rural and Urban

## Abstract

Variations in cancer incidence, mortality and pattern exist in rural and urban areas. Understanding these differences helps in developing targeted cancer prevention and control strategies. However, no previous studies have explored the differences in cancer demographics between the rural and urban areas of Nepal. The data of Kathmandu Valley (urban area) Population-Based Cancer Registry (PBCR) and Rukum (rural area) PBCR were analysed to identify the differences in cancer pattern in rural and urban areas.

The age-adjusted incidence rate (AAR) in Kathmandu was higher than that in Rukum (1.6 times among males and 1.9 times among females). The top two leading sites in males were lungs and stomach in both the regions; however, the rates were higher in Kathmandu. The incidence rate for cancer of the urinary bladder among males in Kathmandu was particularly higher – 4.4 times that of Rukum. In females, the leading site of cancer in Kathmandu was breast, which was eight times higher compared to Rukum, whereas the incidence rate of cervix cancer in Kathmandu is 30% less than in Rukum. The incidence of tobacco-related cancer was found to be higher in Kathmandu compared to Rukum.

These findings reveal the need for different policy priorities for cancer control in the urban versus rural regions of Nepal, based on the different demographics of cancer in the two areas. Similar studies from other regions of Nepal are needed to develop a targeted cancer control strategy.

## Introduction

The burden of cancer is on the rise worldwide. According to GLOBOCAN, an estimated 19.3 million new cancer cases were diagnosed, accounting for 9.9 million death cases in 2020, which has increased from previous years [1]. Among them, around half of the cancer cases and more than half of the cancer deaths were estimated to occur in Asia [1, 2]. Disparities in cancer incidence, patterns and mortality occur not only across countries but also across different regions within the same country. Many studies have reported that the cancer incidence and mortality rates are higher among urban residents than rural residents. Studies also suggest that rural residents are diagnosed in advanced stages, which might influence cancer mortality [3, 4].

B.P. Koirala Memorial Cancer Hospital (BPKMCH) piloted a Population-Based Cancer Registry (PBCR) in 2013 in Nepal [5]. The Nepal Health Research Council (NHRC) initiated the PBCR in January 2018. Currently, Nepal has three PBCRs: the Kathmandu Valley (hereinafter, refers to as Kathmandu) PBCR, Siraha, Saptari, Dhanusha and Mahottari (SSDM) PBCR and Rukum PBCR which covers 9 out of 77 districts of Nepal [6, 7]. While comparing with the estimated 29,218,867 (as per Central Bureau of Statistic) population of Nepal in 2018, the registries in total cover 20.3% of the total population. The population for all registries in 2018 has been estimated based on censuses in 2001 and 2011 [8, 9] in which the Kathmandu PBCR had 3,071,932 (10.5% of the total population), SSDM PBCR had 2,846,036 populations (9.7%) and Rukum PBCR had 221,376 (0.7%) populations [7].

The present study analyses the differences in cancer demographics (incidence, mortality and pattern) in urban and rural areas using data from the Kathmandu and Rukum PBCRs for the first time in Nepal. Kathmandu is one of the most developed areas of the country having necessary infrastructures, including advanced cancer diagnostic and treatment facilities [10] like radio diagnosis and histopathological diagnosis including certain immunohistochemistry panels, radiotherapy, chemotherapy, onco-surgery, palliative treatment etc. On the contrary, Rukum is one of the least developed areas of the country which has no cancer diagnostic and treatment facilities and has poor transportation and communication services. Patients with cancer in Rukum usually travel to larger cities, such as Nepalgunj, Bharatpur, Kathmandu and sometimes to India, for cancer diagnosis and treatment, a distance ranging from 300 to 1,200 km.

## Methods

The Kathmandu PBCR consists of three districts, namely Kathmandu, Bhaktapur and Lalitpur, representing the urban area, whereas the Rukum PBCR comprises two districts, East and West Rukum, predominantly representing the rural and hilly region of the country. The data collection process was primarily active for both registries. The methodology has been explained in previous reports [11, 12].

The data were obtained from multiple sources that included public and private hospitals, pathology laboratories, hospices, Ayurvedic centres, the Social Security and Nursing Division and community (Figure 1).

In the Kathmandu PBCR, data were collected from 28 cancer hospitals and general hospitals where there are cancer diagnostic and treatment facilities, three pathology laboratories, three hospices and two Ayurvedic centres. The other source is Social Security and Nursing Division at the Department of Health Services, as the Government of Nepal provides a subsidy of Nepali Rupees 100,000 (equivalent to approximately USD 1,000) for the treatment of underprivileged cancer patients through the Social Security and Nursing Division. Civil registration is also a major source in any cancer registry; however, in the Nepalese context, civil registration was excluded from the source of data as it did not record the cause of death as cancer. Because some patients visit a cancer hospital, BPKMCH in Bharatpur located about 250 km from Kathmandu, and some patients to India, data were obtained through passive method from these sources as well. In order to obtain data from the communities, the female community health volunteers (FCHVs), community-based health post in-charge and the health coordinators at rural municipalities were trained and mobilised. The primary data collected by FCHVs through home visits were reported to the health posts in-charge, then to the health coordinators by the in-charges and the coordinators to the registry office on a periodic basis. In addition to this, in places with a lower incidence than the estimated incidence rate, trained field enumerators were mobilised to find and collect the information of cancer cases from there (Figure 1).

For the Rukum PBCR, all the diagnostic centres and hospitals in neighbouring districts with the possibility of receiving patients from Rukum were also included as the data sources including 13 hospitals in Kathmandu Valley, BPKMCH in Bharatpur, Nepalgunj Medical College and Teaching Hospital in Nepalgunj. Unlike the Kathmandu Valley PBCR, the major source of data for the Rukum PBCR was community. Trained field enumerators visited the ward chairperson, local leaders, health coordinators/in-charges and the FCHVs to identify the cancer cases in their locality, followed by the household visits for data collection. The data obtained from the community were confirmed by visiting the diagnostic and treatment centres where the patients were treated. However, in some cases due to the lack of proper recording and electronic medical record system, patients’ information was not found. The information obtained from the community was considered in these cases.

All the collected data were checked and verified for completeness and accuracy through the attached relevant patients report (like discharge summary and various investigation reports) at the NHRC office by the trained registry staffs. Besides the data obtained from the community, residence confirmation of the patient was carried out through individual phone calls. The people residing in the selected areas for more than 6 months prior to diagnosis of the disease were considered as residents. Then, the data were entered into CanReg5 software developed by the International Agency for Research on Cancer (IARC) which carried out duplicate checks, check for internal consistency and validity and carried out analysis [13]. For the analysis purpose, the age-wise population of the registry areas in 2018 was estimated based on the population censuses in 2001 and 2011 [8, 9]. Then, the age standard incidence and mortality rate among males and females of all cancer cases were calculated and a comparison was made between the two urban and rural registries at 95% confidence interval (CI) based on Boyle and Parkin’s [14] study.

### Ethical consideration

Ethical approval for the study was obtained from the Ethical Review Board of NHRC. Further formal administrative approval was obtained from each of the sources of data, and while collecting the information of cases through house visits at the community, written informed consent was obtained from the participants.

## Results

The cancer incidence and mortality rate both were found to be higher in the Kathmandu district (urban area) compared to the Rukum district (rural area). The overall AAR of Kathmandu (95.7 per 100,000) is nearly two times higher than in Rukum (54.8 per 100,000). Table 1 shows the comparison of rural and urban cancer incidence rates among males and females separately. The AAR of all cancer sites among males in Kathmandu (95.3 per 100,000) was 1.6 times higher (95% CI: 1.26–2.06) than the incidence rates in Rukum (59.1 per 100,000). Similarly, the AAR of all cancers was 1.9 times higher (95% CI: 1.52–2.40) among females in Kathmandu (98.1 per 100,000) versus females in Rukum (51.4 per 100,000). Although the mortality rates were higher in Kathmandu Valley (male: 36.3 per 100,000, female: 27 per 100,000) compared to Rukum (male: 21.4 per 100,000, female: 25.1 per 100,000), the difference was not as high as it was for incidence rates. However, in both the areas the death cases were under-reported.

Cancers of the lungs and stomach were the commonest cancer among males in both Kathmandu and Rukum (Table 1) with no significant difference in incidences between the two regions. Cancer of the urinary bladder in males was the third most common site of cancer in Kathmandu, the incidence rate of which was 4.4 times (95% CI: 1.60–12.01) higher than that in Rukum. Gallbladder cancer among males was the fourth most common cancer in the urban area and fifth commonest in the rural area. Cancer of the prostate was the fourth most common cancer in Rukum; however, the incidence rates of prostate cancer in Rukum and Kathmandu were the same (3.0 per 100,000).

In females, the commonest cancer sites in the urban area were breast, lungs, cervix uteri, gallbladder and ovary, whereas in the rural areas the commonest cancer sites were cervix uteri, lungs, uterus, ovary and breast. While comparing the common sites in urban and rural areas, the incidence rates of breast cancer in Kathmandu were eight times higher (95% CI: 4.79–13.25) than the incidence rates in Rukum and the incidence rate of gallbladder cancer in females of the urban region (7.4) was 3.1 times higher (95%CI: 1.35-7.06) than the rural region (2.4). The incidences of cancer of the lung and ovary were higher in females in Kathmandu and cancers of the cervix and uterus were higher in Rukum, although these differences were not statistically significant (Table 1).

**Figure 2.**
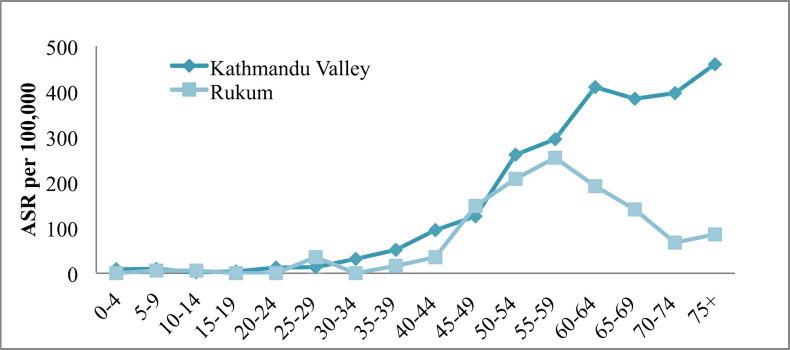
All sites’ age-specific incidence rates (ASR) among females in Kathmandu Valley and Rukum, 2018.

The incidence of cancer increased with increasing age only in Kathmandu (highest incidence in the age group of 70–74 years) but it was inconsistent in Rukum (highest incidence in the age group of 65–69 years) among males (Figure 1). Among females in Kathmandu, the incidence rate increased with age and reached the highest in the age group of 75+ years; however, in Rukum, although the incidence rate increased with age and reached highest at the age group of 55–59 year, it slowly decreased later (Figure 2).

Assessing the data quality indicators, the Kathmandu PBCR recorded 90.5% of the cancer cases as microscopically verified cases, whereas the Rukum PBCR recorded only 56.3% of the cases through microscopic verification. Higher the rate of microscopic verification of cases, higher the quality of registry [15]. The cases recorded through verbal information were high in Rukum (33.3%), and this refers to the cases obtained through community from house visits, where the patient and/or relatives provided information about cancer verbally and we could not obtain reports from the patients and were not able to trace back the patient’s records in the hospitals, due to the unscientific ways of record-keeping at many sources. The Death Certificate Only (DCO) cases were low in both areas, i.e. 1.4% in Kathmandu Valley and 1.1% in Rukum district. Lower the proportion of DCO cases, the higher the quality of the registry is considered. To our knowledge, very few deaths due to cancer had occurred in Rukum; hence, a meaningful comparison of mortality trends in the two regions could not be made.

## Discussion

While comparing the data of the two registries, we found substantial differences in the demographics of cancer cases in the two regions; therefore, suggesting a differential approach to tackling cancer is needed in the urban versus rural areas of Nepal. These data also support the efforts to expand PBCR to encompass every district in Nepal as a priority policy intervention [10].

**Figure 3.**
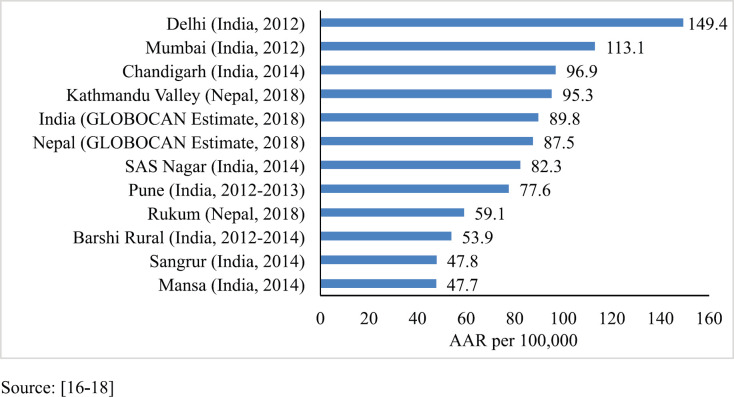
Comparison of AAR of all sites with neighbouring areas (males).

**Figure 4.**
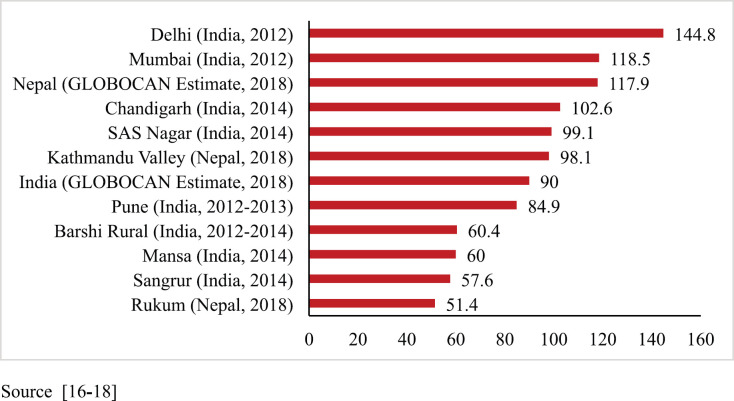
Comparison of AAR of all sites with neighbouring areas (females).

The PBCR data of Kathmandu and Rukum were compared with the estimated rate given by GLOBOCAN for Nepal in 2018 and with the urban and rural areas of the neighbouring country India that have similar context. For males, compared to the 2018 estimate by GLOBOCAN for Nepal (87.5 per 100,000) [16], the rate of cancer incidence in Kathmandu was higher but that in rural Nepal (Rukum) was lower. However, these rates were comparable to those of the urban and rural registries of India, respectively [17, 18] (Figure 3). For females, the incidence rates were much lower in both urban and rural registries as compared to the 2018 estimate given by GLOBOCAN for Nepal (117.9 per 100,000) [16]. The AAR in Kathmandu among females is also lower than some of the urban areas of India; however, the AAR in Rukum among females is comparable to the rural areas of India [17, 18] (Figure 4).

Our study found that there was a significant difference in the cancer incidence rates between the urban and rural areas of Nepal. In Kathmandu, the AAR was nearly two times (1.6 times in males and 1.9 times in females) higher than that in Rukum. Similar differences in the rural and urban areas have been reported in other countries, including India. [3, 4, 19–21]. The higher rate of cancer incidence in urban region compared to rural is multifactorial, but is not limited to, higher prevalence of risk factors of cancer like sedentary lifestyle, smoking, environmental pollutions, better diagnostic opportunities, better socio-economic status and possibly genetic/ethnic differences in population [4]. The World Health Organisation (WHO)’s non-communicable disease STEPwise approach to surveillance survey also found that one-fourth of the people in metropolitan city were overweight and obese compared to only 18% in rural municipalities [22], and it is now well known that obesity is a risk factor for several cancers [23].

In both rural and urban areas, the incidence of cancer in males and females has started increasing after the age of 35. However, the incidence rate in rural areas was highest among the earlier age group (65–69 years in males and 55–59 years in female) compared to urban areas (above 70 years in both male and female). This could partially be explained by the lack of access to diagnostic facilities among the elderly population in rural Nepal compared to the urban [4].

Some unique differences among the common cancer types between urban and rural Nepal include the higher proportion of cervical cancers among women in Rukum. Cervical cancer incidence and mortality are directly related to the socio-economic status [24]. Increased awareness, cervical cancer screening opportunities and Human Papilloma Virus vaccination should be the cornerstone of any cancer control policy in rural Nepal. Mobile-based technologies have shown potential in early detection of cervical cancer in rural Africa [25], a model that could potentially be replicated in Nepal in the spirit of global south-to-south collaboration.

The increased incidence of cancer of the urinary bladder in either sex in Kathmandu should be investigated for potential causes such as high arsenic exposure. Various studies have shown that the groundwater of Kathmandu Valley exceeds the WHO’s recommended arsenic value [26, 27] and groundwater in the valley is one of the major sources of water, whereas in Rukum people rely on the natural surface water. In addition, the reason for increased incidence of gallbladder in Kathmandu is also intriguing. Gallbladder cancer is a relatively rare cancer globally [28], but the incidence is higher in areas near Nepal such as North and Northeast India [29-31]. One potential hypothesis is the increased incidence of gallstones. The high incidence in Kath mandu as reported in our study needs more collaboration for cancer control and treatment exercises with nearby regions, such as Kerala, to understand this phenomenon better.

As it was the first year of the registry, there were some difficulties in both urban and rural registries in finding the cancer cases and collecting information. The absence of scientific record-keeping systems at the healthcare facilities was a major challenge in both urban and rural registries. Initially, some of the data sources denied sharing the cancer data and both registries faced difficulties in collecting information. However, the problem was solved through a circular from the Ministry of Health and Population (MoHP) and the Ministry of Federal Affairs and General Administration, as well as numerous coordination meetings. In the rural registry, the lack of cancer diagnostic and treatment facilities has caused more challenges to capture all the cases/information as the patients might go for diagnosis and treatment at diverse locations. Similarly, while collecting data from the community, hiding information due to the fear of social stigma remained another challenge.

There are certain limitations to our study. Being the first year of the registry, some possible under registration could not be ruled out, especially in the rural areas, which is one of the common problems faced by any registry during the first few years [32]. The number of mortality events recorded was also low precluding any meaningful comparisons for mortality trends. However, as the registry continues, under-registration will be minimised. The rural and urban registry data will be used for planning cancer control activities as well as for the development of infrastructure, such as early diagnosis and treatment in rural areas.

## Conclusion

Our comparative analysis of cancer demographics using population-based cancer registries of Kathmandu and Rukum has revealed important differences in cancer incidence and patterns between urban and rural Nepal. These data are important for authorities such as the federal and the provincial government to understand and plan for cancer control strategies such as screening campaigns, diagnostic needs and treatment facilities. Such a comparison has been made possible only due to the establishment of a PBCR. This study attests to the need to expand this PBCR to cover every district in Nepal so that cancer control efforts can be tailored to local needs.

## List of abbreviations

AAR: Age-Adjusted Incidence Rate
BPKMCH: B.P. Koirala Memorial Cancer Hospital
CI: Confidence Interval
DCO: Death Certificate Only
FCHVs: Female Community Health Volunteers
IARC: International Agency for Research on Cancer
NHRC: Nepal Health Research Council
PBCR: Population-Based Cancer Registry
WHO: World Health Organisation

## Conflicts of interest

Dr Gyawali reports receiving consulting fees from Vivio Health. Other authors report no conflicts of interest.

## Authors’ contributions

Ranjeeta Subedi: conceptualisation, methodology, formal analysis, data curation, writing the original draft and project administration. Atul Budukh: conceptualisation, methodology, validation, review of the article and supervision. Sandhya Chapagain: conceptualisation, methodology, validation, review of the article and supervision. Pradip Gyanwali: resources, review of the article and supervision. Bishal Gyawali: conceptualisation, writing the original article and supervision. Kopila Khadka: Data curation and formal analysis. Chanda Thakur: data curation and formal analysis. Uma Dahal: data curation and formal analysis. Rajesh Dikshit: conceptualisation, methodology, validation, review of the article and supervision. Anjani Kumar Jha: conceptualisation, methodology, resources, validation, review of the article and supervision. Meghnath Dhimal: conceptualisation, methodology, writing the original draft and supervision

## Figures and Tables

**Figure 1.**
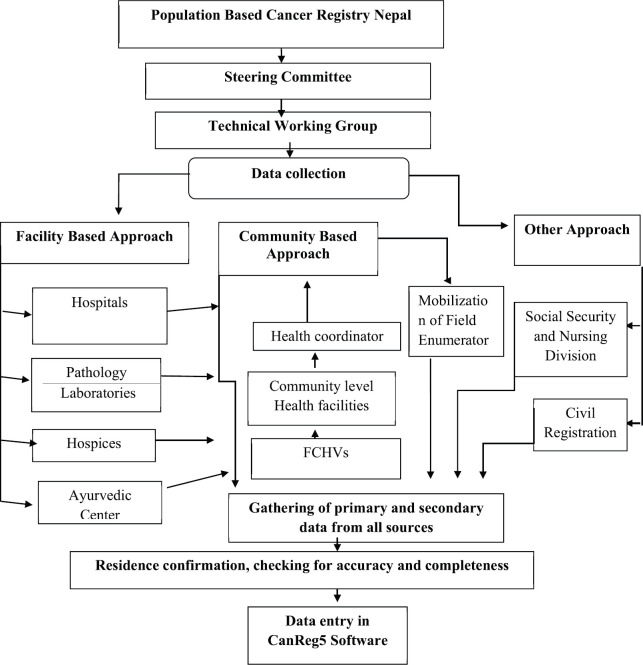
Flowchart of the population-based cancer registry, Nepal (Source: [11]).

**Figure 1.**
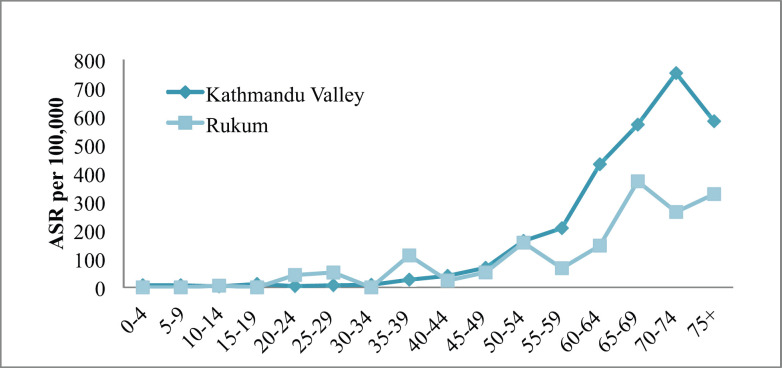
All sites’ age-specific incidence rates (ASR) among males in Kathmandu Valley and Rukum, 2018.

**Table 1.** Comparison of the cancer incidences in the urban and rural registries of Nepal (2018).

Sites	Rukum (AAR)	Kathmandu (AAR)	Rate ratio	95% CI	
All sites of cancer: Male	59.1	95.3	1.6	1.26–2.06	Significant
All sites of cancer: Female	51.4	98.1	1.9	1.52–2.40	Significant
Stomach cancer: Male	7.3	8.5	1.2	0.50–2.71	Not Significant (NS)
Stomach cancer: Female	2.6	4.2	1.6	0.52–4.98	NS
Gallbladder cancer: Male	2.9	5.5	1.9	0.66–5.44	NS
Gallbladder cancer: Female	2.4	7.4	3.1	1.35–7.06	Significant
Lung cancer: Male	13.2	18.1	1.4	0.79–2.39	NS
Lung cancer: Female	5.2	10.4	2	0.95–4.19	NS
Breast cancer	2.7	21.5	8	4.79–13.25	Significant
Ovary cancer	3.2	5.5	1.7	0.69–4.30	NS
Cervix cancer	13.2	8.7	0.7	0.33–1.32	NS
Uterus cancer	3.9	0.5	0.1	0.01–1.89	NS
Prostate cancer	3	3	1	–	Same No difference
Thyroid cancer: Male	4.2	1	0.2	0.03–2.10	NS
Bladder cancer: Male	1.3	5.7	4.4	1.60–12.01	Significant

Analysis based on Boyle and Parkin [14]
